# Melatonin Reduces Angiogenesis in Serous Papillary Ovarian Carcinoma of Ethanol-Preferring Rats

**DOI:** 10.3390/ijms18040763

**Published:** 2017-04-11

**Authors:** Yohan Ricci Zonta, Marcelo Martinez, Isabel Cristina C. Camargo, Raquel F. Domeniconi, Luiz Antonio Lupi Júnior, Patricia Fernanda F. Pinheiro, Russel J. Reiter, Francisco Eduardo Martinez, Luiz Gustavo A. Chuffa

**Affiliations:** 1 Department of Anatomy, Institute of Biosciences, São Paulo State University (UNESP), Botucatu-SP 18618-970, Brazil; yohanzonta@hotmail.com (Y.R.Z.); rdomeniconi@ibb.unesp.br (R.F.D.); lupijr@ibb.unesp.br (L.A.L.J.); pinheiro@ibb.unesp.br (P.F.F.P.); martinez@ibb.unesp.br (F.E.M.); 2 Department of Morphology and Pathology, Universidade Federal de São Carlos (UFSCar), São Carlos-SP 13565-905, Brazil; martinez@ufscar.br; 3 Department of Biotechnology, School of Sciences, Humanities and Languages, São Paulo State University (UNESP), Assis-SP 19806-900, Brazil; camargo@assis.unesp.br; 4 Department of Cellular and Structural Biology, University of Texas Health Science Center at San Antonio (UTHSCSA), San Antonio, TX 78229, USA; reiter@uthscsa.edu

**Keywords:** ovarian cancer, melatonin, angiogenesis, VEGF (vascular endothelial growth factor), VEGFR (VEGF receptor), hypoxia-inducible factor (HIF)-1α

## Abstract

Angiogenesis is a hallmark of ovarian cancer (OC); the ingrowth of blood vessels promotes rapid cell growth and the associated metastasis. Melatonin is a well-characterized indoleamine that possesses important anti-angiogenic properties in a set of aggressive solid tumors. Herein, we evaluated the role of melatonin therapy on the angiogenic signaling pathway in OC of an ethanol-preferring rat model that mimics the same pathophysiological conditions occurring in women. OC was chemically induced with a single injection of 7,12-dimethylbenz(a)anthracene (DMBA) under the ovarian bursa. After the rats developed serous papillary OC, half of the animals received intraperitoneal injections of melatonin (200 µg/100 g body weight/day) for 60 days. Melatonin-treated animals showed a significant reduction in OC size and microvessel density. Serum levels of melatonin were higher following therapy, and the expression of its receptor MT1 was significantly increased in OC-bearing rats, regardless of ethanol intake. TGFβ1, a transforming growth factor-beta1, was reduced only after melatonin treatment. Importantly, vascular endothelial growth factor (VEGF) was severely reduced after melatonin therapy in animals given or not given ethanol. Conversely, the levels of VEGF receptor 1 (VEGFR1) was diminished after ethanol consumption, regardless of melatonin therapy, and VEGFR2 was only reduced following melatonin. Hypoxia-inducible factor (HIF)-1α was augmented with ethanol consumption, and, notably, melatonin significantly reduced their levels. Collectively, our results suggest that melatonin attenuates angiogenesis in OC in an animal model of ethanol consumption; this provides a possible complementary therapeutic opportunity for concurrent OC chemotherapy.

## 1. Introduction

Ovarian cancer (OC) is the most lethal gynecological carcinoma among women and it exhibits poor prognosis when diagnosed at an advanced stage (with a <50% survival rate at five years) [1]. Even with the current therapies, including chemotherapy and surgical removal, patients in stage III or IV have only a 20% survival rate [2,3]. To date, early-stage OC has no apparent symptoms, and no significant screening method is yet available [4]. 

The influence of chronic alcoholism on the acquisition and progression of many types of cancer is indisputable. Alcohol abuse is associated with several changes in the reproductive system, such as including alterations of menopause, anovulation, infertility, and even cancer [5,6]. Moreover, acetaldehyde originating from ethanol oxidation acts as a co-carcinogenic agent through association with DNA, and by promoting cross-links between pairs of bases G:A and A:T [7,8]. Notably, we have developed a useful ethanol-preferring rat model that strictly reflects the aspects of serous papillary OC after chemical induction with a carcinogen [9,10]; this model provides an appropriate histological and molecular background that is needed to evaluate new chemoprotective compounds.

Angiogenesis is a complex and determining event for tumor growth and metastatic spread [11]. Its dynamic evolution is dependent on pro- and anti-angiogenic molecules. Vascular endothelial growth factor (VEGF) is highly expressed in tumor cells under numerous conditions such as hypoxia, acidosis, mechanical stress, and an imbalance of tumor suppressor genes. The VEGF receptor (VEGFR) is expressed on the surface of OC cells, and is associated with the development of malignant ascites and tumor progression [12]. Expressions of VEGFR1 and VEGFR2 are significantly elevated in OC cells; specifically, VEGFR2 is more functionally active in decreasing microvascular density [13]. The synthesis of VEGF and its receptors is regulated by the hypoxia-inducible factor 1-alpha (HIF-1α), a heterodimer sensitive to the fluctuations in oxygen levels [14]. Under hypoxia, HIF-1α is stabilized and enters the nucleus, where it binds to the hypoxia responsive elements (HRE) to activate the transcription of many genes related to tumor aggressiveness and chemoresistance [15,16]. While VEGF-targeted therapies (e.g., bevacizumab—a monoclonal humanized antibody against VEGF) showed positive results in clinical practice, its major effects are related to the optimal dose, duration of treatment, and specific OC subtype [17]. Importantly, the functional significance of the VEGF/VEGFR signaling pathway is not completely understood in OC, and further studies are required. Recently, the transforming growth factor-β (TGF-β) was found to be associated with the occurrence, progression, and metastasis of OC [18], and TGF-β production may represent an important strategy of tumor escape, favoring angiogenesis and increasing the interaction between OC cells and components of the extracellular matrix [19].

Melatonin (*N*-acetyl-5-methoxytryptamine) is an indoleamine secreted in a circadian manner by the pineal gland with maximal production during the night [20]. It has remarkable functional versatility, acting as an antioxidant, immunomodulatory, and oncostatic agent in many cancer types including OC [4,21,22,23]. At pharmacological concentrations, melatonin has anti-angiogenic activity [24,25]. Recent publications have reported that melatonin significantly reduced the expression of VEGF and HIF-1α in breast and colon cancer in both in vitro and in vivo studies [26,27], thus controlling neovascularization. To date, no study has elucidated the cross-talk between melatonin and angiogenesis in OC.

Herein, we investigated the influence of long-term melatonin therapy on the angiogenic process (VEGF/VEGFR/HIF-1α pathway) in serous papillary ovarian carcinoma of an ethanol-preferring rat model. We further tested whether melatonin is effective in reducing angiogenesis in OC of animals exposed to ethanol.

## 2. Results

### 2.1. Anatomopathological Analysis of OC (Ovarian Cancer) and Melatonin Levels

**Figure 1:**
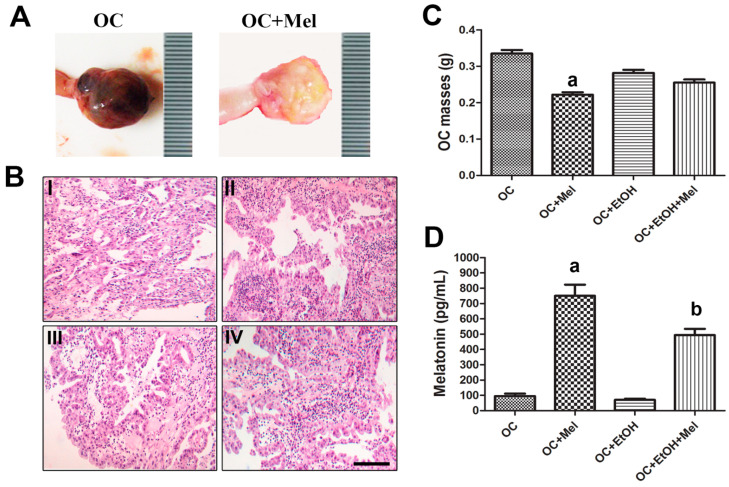
Development and treatment of OC (Ovarian Cancer). (**A**) Anatomopathological specimens of the untreated and melatonin-treated OCs; (**B**) micrographs of the OCs showing serous papillary architecture in OC (**I**), OC + Mel (melatonin) (**II**), OC + EtOH (ethanol) (**III**), and OC + EtOH + Mel (**IV**) groups. Bar = 20 µm; (**C**) OC mass was measured at the end of treatments, ^a^ *p* < 0.05 vs. OC; (**D**) plasma melatonin levels (pg/mL) after the last treatment dose, ^a,b^ *p* < 0.05 vs. OC and OC + EtOH, respectively. *n* = 15 animals/group.

The association between EtOH (ethanol) intake with DMBA led to a high incidence of OC after 180 days of age (93%), and melatonin treatment showed a slight reduction on OC incidence rate (by 80% and 86.6% in OC + Mel (melatonin) and OC + EtOH + Mel groups, respectively). After treatment, the abdominal–pelvic cavity was opened and the left ovaries of the OC animals displayed solid masses with neovascularization spots—different from the ovaries of the OC + Mel group, which exhibited soft and clear masses with no adhesions (Figure 1A). Furthermore, histopathological analyses of the OC tissue confirmed the presence of serous papillary carcinoma with cellular atypia and exophytic architecture mixed with invasive gland-like neoplastic structures or tufts back-to-back (Figure 1B). Because the OC subtype was maintained, no microscopic changes were observed following EtOH consumption and melatonin therapy (Figure 1B(I–IV)). We further investigated the effects of melatonin on the OC masses and, notably, long-term melatonin significantly reduced the OC size by about 36% (Figure 1C). To validate the treatments, plasma melatonin was measured at the end of the dark phase. The animals treated with melatonin (OC + Mel and OC + EtOH + Mel groups) showed higher levels of circulating melatonin than the other groups (Figure 1D).

### 2.2. Melatonin Therapy Reduced the Vessel Density in OC

**Figure 2:**
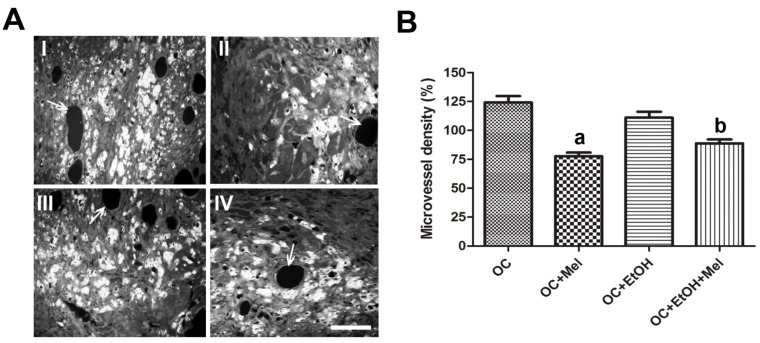
Analysis of the microvessel density. (**A**) Representative histological images showing the pattern of vascularization in OC (**I**), OC + Mel (**II**), OC + EtOH (**III**), and OC + EtOH + Mel (**IV**). Bar = 20 µm. (**B**) Quantitative analysis of microvessel density (%) was achieved by counting positive vessels in the field (white arrows). ^a,b^ *p* < 0.05 vs. OC and OC + EtOH, respectively. *n* = 15 animals/group.

To confirm the effect of melatonin on tumor neovascularization, we performed a quantification of microvessel density (MVD) in seriated sections of OC tissue. The melatonin-treated animals showed a significant reduction of MVD compared to the controls, regardless of EtOH intake (*p* < 0.05; Figure 2A,B).

### 2.3. MT1 (Type 1 Melatonin Receptor) Is Upregulated by Melatonin in OC While Transforming Growth Factor (TGF)-β1 Levels Are Downregulated

**Figure 3:**
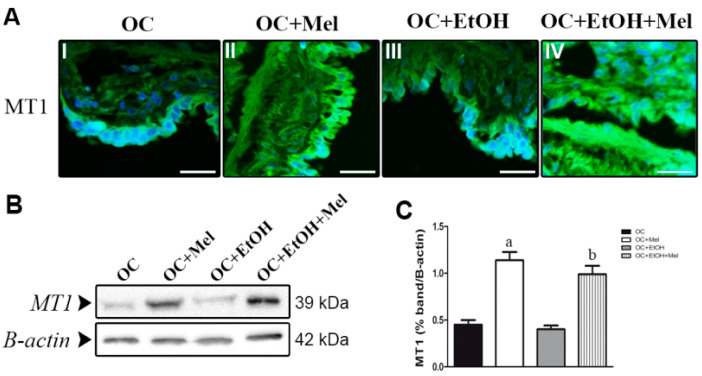
Immunofluorescence localization and Western blot analysis of MT1 (type 1 melatonin receptor) in serous papillary OC. (**A**) Merged images of the immunofluorescence of MT1 and DAPI (4′,6-Diamidino-2-Phenylindole ) nuclear staining in OC (**I**), OC + Mel (**II**), OC + EtOH (**III**) and OC + EtOH + Mel (**IV**) groups; (Alexa fluor^®^ 488, Bar = 20 µm, negative controls were used); (**B**) Representative MT1 profile of extracts (70 µg proteins) pooled from seven samples/group (left panel); (**C**) extracts obtained from individual animals were used for densitometric analysis of the MT1 levels following normalization to the β-actin. Data are expressed as the mean ± SD (*n* = 7). ^a^ *p* < 0.05 vs. OC; ^b^ *p* < 0.05 vs. OC + EtOH.

To investigate whether melatonin treatment alters the expression of its receptor, we measured MT1 intensity through immunofluorescence assays. MT1 was significantly increased by long-term melatonin therapy in epithelial cells of OC (Figure 3A(I,II); the fluorescence level was augmented from 37% ± 9.2% (OC group) to 86% ± 13.4% (OC + Mel group)). Even in EtOH-drinking animals, melatonin therapy efficiently resulted in upregulation of MT1 (Figure 3A(III,IV); the fluorescence level increased from 32.8% ± 15.7% (OC + EtOH) to 79% ± 10.6% (OC + EtOH + Mel)). MT1 levels were further confirmed in serous papillary OC tissues, and immunoblots showed that melatonin therapy upregulated its own receptor MT1 in the OC + Mel (2.58-fold increased vs. OC; Table 1, Figure 3B,C) and OC + EtOH + Mel (2.34-fold increased vs. OC + EtOH; Table 1, Figure 3B,C) rats.

**Figure 4:**
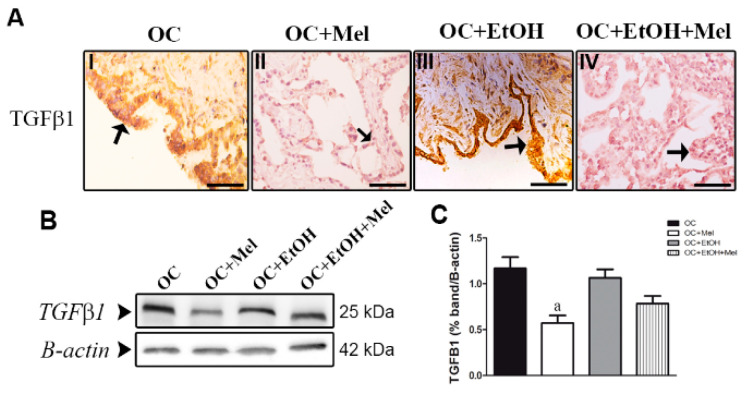
Immunohistochemical localization and western blot analysis of TGFβ1 in serous papillary OC. (**A**) The immunoreaction of TGFβ1 in the epithelial cells of OC was stronger in the OC (**I**) and OC + EtOH (**III**) groups compared to a weak reaction observed in OC + Mel (**II**) and OC + EtOH + Mel (**IV**) animals (black arrows). Bar = 20 µm. Negative controls were used. (**B**) Representative TGFβ1 profile of extracts (70 µg proteins) pooled from seven samples/group (left panel). (**C**) Extracts obtained from individual animals were used for densitometric analysis of the proteins following normalization to house-keeping protein β-actin. All results are expressed as the mean ± SD (*n* = 7). ^a^ *p* < 0.05 vs. OC.

TGFβ1, an important factor associated with tumor migration and invasiveness, was only downregulated following melatonin therapy alone. In the OC + Mel group, the epithelial cells of the papillae and stromal cells showed a weak signal for TGFβ1 (Table 1, Figure 4A(I,II)), different from the OC + EtOH + Mel group, which showed a moderate signal (Table 1, Figure 4A(III,IV)). Furthermore, the expression of TGFβ1 was significantly reduced after melatonin therapy (1.16-fold reduced vs. OC; Figure 4B,C).

### 2.4. Melatonin Downregulates VEGF (Vascular Endothelial Growth Factor) and VEGFR2 (VEGF Receptor 2), Even in the Presence of Ethanol

**Figure 5:**
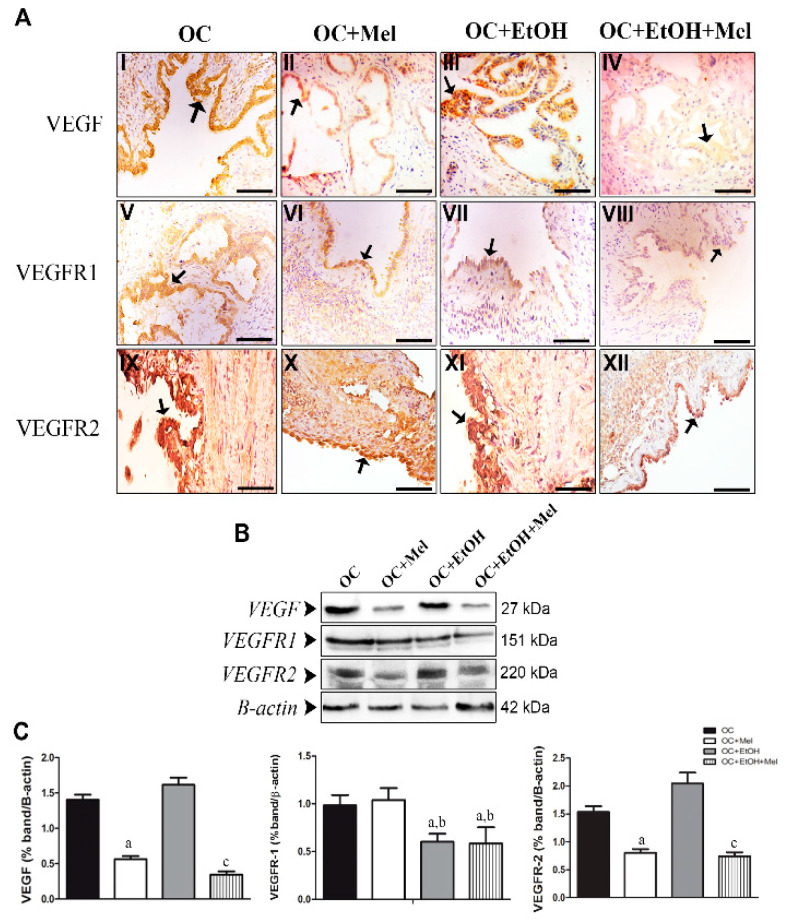
Immunohistochemical localization and western blot analysis of VEGF, VEGFR1, and VEGFR2 in serous papillary OC. (**A**) The immunoreaction of VEGF was moderate to high in the OC (**I**) and OC + EtOH (**III**) groups, while the groups OC + Mel (**II**) and OC + EtOH + Mel (**IV**) showed a weak reaction after melatonin treatment (black arrow). The immunoreactions of VEGFR1 varied from weak to moderate in the surface epithelium of the OC (**V**) and OC + Mel (**VI**) animals, and only a weak reaction was notable in the OC + EtOH (**VII**) and OC + EtOH + Mel (**VIII**) groups (black arrow). A strong reaction to VEGFR2 was present in the papillae epithelium of the OC (IX) and OC + EtOH (**XI**) groups, and melatonin treatment led to weak or even absence of VEGFR2 immunostaining in the OC + Mel (**X**) and OC + EtOH + Mel (**XII**) animals (black arrows). Bar = 20 µm. Negative controls were used. (**B**) Representative profile of the VEGF, VEGFR1, and VEGFR2 levels obtained from protein extracts (70 µg) pooled from seven samples/group (upper panel). (**C**) Extracts obtained from individual animals were used for densitometric analysis of the proteins following normalization to house-keeping protein (β-actin). Data are expressed as the mean ± SD (*n* = 7). ^a^ *p* < 0.05 vs. OC; ^b^ *p* < 0.05 vs. OC + Mel; ^c^ *p* < 0.05 vs. OC + EtOH.

We investigated the VEGF/VEGFR signaling pathway in OC tissues. Notably, VEGF was downregulated by melatonin in epithelial cells (1.40-fold reduction vs. OC; Table 1, Figure 5A(I,II),B,C). Similarly, the combination of EtOH with melatonin promoted a significant reduction in the VEGF levels (2.19-fold reduction vs. OC + EtOH; Table 1, Figure 5A(III,IV),B,C). Unexpectedly, while VEGFR1 was unchanged by melatonin therapy (*p* > 0.05; Figure 5A(V,VI),B,C), their levels were downregulated by either EtOH alone or the combination of EtOH and melatonin (Table 1, Figure 5A(VII,VIII),B,C), showing that EtOH consumption is responsible for VEGFR1 reduction. Conversely, melatonin therapy significantly reduced VEGFR2 levels in serous papillary OC, regardless of EtOH intake (Table 1, Figure 5A(IX–XII)). Also, melatonin alone (1.48-fold reduction vs. OC; Figure 5B,C), and the combination of EtOH and melatonin (1.77-fold reduction vs. OC + EtOH; Figure 5B,C) led to downregulation of VEGFR2. 

### 2.5. Hypoxia-Inducible Factor (HIF)-1α Is Downregulated by Melatonin Only in the Presence of EtOH Intake

**Figure 6:**
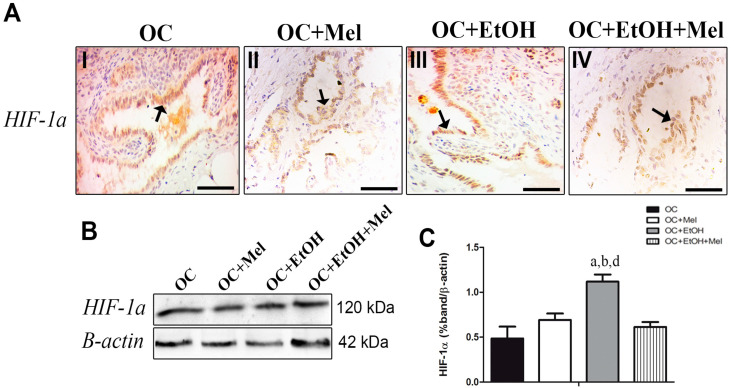
Immunohistochemical localization and Western blot analysis of HIF-1α in serous papillary OC. (**A**) The immunoreaction of HIF-1α in the epithelial cells of OCs was moderate to weak in the OC (**I**) and OC + Mel (**II**) groups compared to a strong reaction observed in OC + EtOH (**III**) animals. In OC + EtOH animals, melatonin treatment reduced the HIF-1α intensity closely to control levels (**IV**) (black arrows). Bar = 20 µm. Negative controls were used; (**B**) Representative HIF-1α profile of extracts (70 µg protein) pooled from seven samples/group (left panel); (**C**) Extracts obtained from individual animals were used for densitometric analysis of the proteins following normalization to house-keeping protein β-actin. Data are expressed as the mean ± SD (*n* = 7). ^a^ *p* < 0.05 vs. OC; ^b^ *p* < 0.05 vs. OC + Mel; ^d^ *p* < 0.05 vs. OC + EtOH + Mel.

Although melatonin therapy seemed to slightly reduce HIF-1α immunostaining in the epithelial cells of OC + Mel and OC + EtOH + Mel groups (Table 1, Figure 6A(I–IV)), it was only significantly reduced in the OC + EtOH + Mel group (0.74-fold reduced vs. OC + EtOH; Figure 6B,C). EtOH consumption resulted in higher levels of HIF-1α compared to other groups and melatonin therapy efficiently restored these levels to near those in the OC rats.

### 2.6. Melatonin and Ethanol Differentially Modulated the VEGF/VEGFR/HIF-1α Pathway

**Figure 7:**
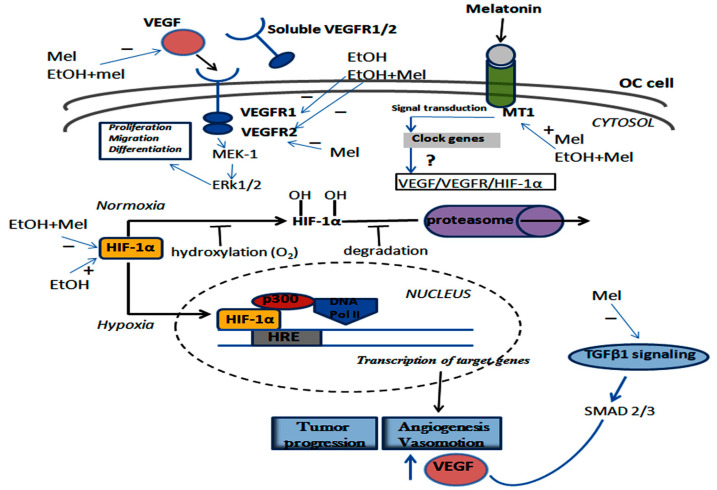
Schematic representation of the VEGF/VEGFR/HIF-1α signaling pathway and the effects of melatonin treatment leading to the activation or repression of downstream molecules in OC cell. This activation seems to be mediated by MT1 and possibly via clock genes. Intracellular signaling results in the phosphorylation cascade and, under normoxia, HIF-1α is degraded via the proteasome pathway. Otherwise, under hypoxia condition, the stabilized HIF-1α is translocated to the nucleus to promote the transactivation of target genes related to tumor progression/differentiation, angiogenesis, and vasomotion. VEGF, VEGFR2, HIF-1α, and TGFβ1 are downregulated by Mel and/or the association EtOH+Mel. Melatonin therapy also positively regulated MT1 expression in OC.

Figure 7 summarizes the positive and negative regulatory effects that are influenced by either melatonin therapy or EtOH intake on angiogenic process in the serous papillary OC cell. In general, while EtOH upregulated VEGF and VEGFR2, treatment with melatonin efficiently suppressed the VEGF/VEGFR pathway resulting in reduced neovasculogenesis in OC. A part of this signaling response is thought to be mediated by the MT1 activation.

## 3. Discussion

Angiogenesis plays a critical role in tumor aggressiveness, and a number of anti-angiogenic agents have been tested in clinical trials, including in OC patients [28]. Although most investigations have demonstrated the prognostic value of VEGF in OC samples such as in surgical tissue, serum, and ascites, these results are somewhat inconsistent [29]. In an attempt to explore the effects of melatonin in an in vivo model of OC, we chemically induced OC in ethanol-preferring rats. This model has been shown to be appropriate for the development of aggressive OC subtypes similar to those occurring in women [9].

As uncovered in our previous studies [4,30], melatonin treatment led to an increase of its circulating levels in both melatonin-treated groups, and was sufficient to reduce OC volume and mass, even in the presence of EtOH. The effects of melatonin on cell proliferation and apoptosis have been studied in vitro, basically using the serous papillary human OC cell line (SKOV-3) [31]. In pharmacological concentrations (0.5–2 mM), melatonin induced caspase-3 activation and cleavage of poly-(ADP-ribose) polymerase (PARP), resulting in the control of MAPK (Mitogen Activated Protein Kinase) phosphorylation. In this regard, our previous work reported that long-term melatonin therapy downregulated survivin levels and upregulated p53, BAX, and active caspase-3, identifying a possible pathway by which melatonin promotes cell apoptosis in an in vivo model of OC [32].

We have observed that melatonin significantly reduced the levels of VEGF, VEGFR2, and HIF-1α regardless of EtOH consumption. In addition, melatonin alone lowered by half the levels of TGF-β1 compared to untreated OC-bearing rats. These effects seem to be mediated through its receptor MT1. Although no changes were observed in the histological aspects of the serous papillary OC, long-term melatonin treatment promoted anti-angiogenic activity to this tumor subtype as demonstrated by a significant reduction in the microvessel density. Considering this function, melatonin could be a chemical strategy to limiting neovascularization in OC tissue.

Melatonin exerts antitumor effects through a variety of important mechanisms of action, making it difficult to determine which of these specific actions is most significant [33]. In a rodent mammary gland carcinoma model, chemoprevention with melatonin added to water (20 µg/mL) decreased tumor frequency and cell proliferation, in addition to having pro-apoptotic and anti-angiogenic effects [34]. MT1 activation seems to be responsible for the oncostatic role of melatonin, which results in inhibition of cAMP (cyclic adenosine monophosphate) and related protein kinases [35,36]. This regulatory activity can negatively alter the expression of genes involved in angiogenesis and migration pathways [37]. The expression of MT1 was higher in the surface of OC cell following melatonin therapy. Supporting this finding, treatment with exogenous nanomolar levels of melatonin upregulated MT1 expression in ovarian cancer SKOV-3 and OVCAR-3 cell lines [38], thus demonstrating a potential role of MT1 involvement in the regulation of downstream molecules related to the angiogenic process.

TGF-β1 is overexpressed in cancer tissue, blood, and peritoneal fluid and may contribute to OC progression and metastasis, in particular due to the regulation of the epithelial-to-mesenchymal transition [19]. Furthermore, TGF-β1 stimulates matrix metalloproteinase secretion, thereby promoting OC cell invasion [39]. Several clinical studies report that TGF-β1 and TGF-β1-binding protein mRNAs are elevated in OC tissue and in ascites fluid [19,40]. Although melatonin has been shown to activate the TGF-β1 pathway related to growth inhibition of breast cancer cells [41], we observed an antagonizing effect of melatonin on TGF-β1 and VEGF levels in the course of OC treatment. Together, these results indicated a favorable use of melatonin to protect against OC angiogenesis and metastasis.

A recent meta-analysis in woman with early and advanced stage OC found that both serum (385 patients) and tissue (638 patients) studies revealed a negative correlation between VEGF level and poor progression-free survival or overall survival [29]. In epithelial OC, VEGF is involved in tumor progression and lymphatic metastasis, and VEGFR is aberrantly activated in OC subsets [42,43]. After VEGFR activation, cancer stem cells undergo differentiation, which in turn enhances survival, proliferation, migration, and invasion [44]. Herein, melatonin efficiently attenuated VEGF at the protein level. Strengthening this finding, other studies documented that higher concentrations of melatonin reduce the levels of VEGF mRNA and protein in human pancreatic carcinoma cells, prostate, colon, and breast cancer cells [26,45,46,47]. Conversely, VEGFR1 was profoundly reduced in EtOH-consuming animals with or without melatonin treatment. In particular, VEGFR1 was only downregulated after EtOH intake. Reinforcing these data, it has been shown that EtOH disrupts VEGFR expression and activation of endothelial cells in reproductive tissues [48]. Although alcohol has many adverse effects in tumor development and progression, chronic EtOH consumption was associated to a reduced angiogenic signaling by lowering VEGFR1 levels in OC tissue. Melatonin therapy significantly downregulated VEGFR2, regardless of EtOH intake. VEGFR2 is the isoform predominantly expressed in malignant OC tissue [49], and treatment with anti-VEGFR2 drugs decreased OC cell migration and invasion [50].

The VEGF/VEGFR2 signaling between endothelial cells and tumor cells is one of the most representative systems for tumor-associated angiogenesis [51]; long-term melatonin therapy significantly attenuated this signaling in OC, possibly indicating a dual function of melatonin in inhibiting paracrine pathways created between tumor cells and the vessel endothelium. Consistent with our results, a recent study demonstrated that melatonin reduced the expression of VEGFR2 in breast cancer cells (MCF-7 and MDA-MB-231 cell lines), and also in mammary tumors of athymic nude mice [27,52].

Angiogenesis occurs in response to conditions of low nutrients and oxygen levels, as modulated by HIF-1α [53]. This process is complex and highly dependent on pro- and anti-angiogenic factors. In fact, HIF-1α is critical for the regulation of VEGF gene transcription [54], although VEGF can be controlled by some oncogenes and growth factors. In our study, we found that melatonin did not alter the levels of activated HIF-1α in OC tissue, thus revealing a distinct response in reducing VEGF levels different from a transcriptional regulation by HIF-1α. These data provide clues for understanding new mechanisms related to melatonin-mediated inhibition of angiogenesis in OC, and might aid in uncovering novel combination therapies to control tumor growth. Recently, a number of mechanisms underlying melatonin’s effects on the levels of HIF-1α and VEGF have been discussed, such as via its antioxidant capacity [23,55], functional alteration of the ubiquitin ligase, von Hippel–Lindau (VHL) protein [56], changes in particular microRNA, and others [57]. While some hypotheses have been provided, the mechanisms regulating shifts in the balance of angiogenic activators and regulators remain inconclusive for OC.

The current results support an interesting relationship involving VEGF, VEGFRs, and HIF-1α in OC cell. In this context, melatonin differentially modulated this angiogenic pathway, contributing to a slow growing of the tumors. In addition, melatonin has been proven to significantly reduce OC size and microvessel density, which may adversely impact tissue perfusion, and subsequently, the tumor development. Besides acting on tumor cells, melatonin also exhibits a direct anti-angiogenic activity by inhibiting the proliferation and migration of endothelial cells, as observed here as indicated by low microvessel density. Through its effects, we hypothesized that melatonin could serve as an effective agent for hypoxic adaptation and inhibition of angiogenesis in aggressive tumors (e.g., ovarian tumors), providing a solid foundation for the use of melatonin in basic and preclinical studies. Furthermore, the administration of melatonin is well tolerated, causing no local or systemic toxicity even after a longer period of treatment.

## 4. Materials and Methods

### 4.1. Animals

Sixty UChB rats (a model of ethanol-preferring rat that has been developed by selective inbreeding), 65 days old and weighing approximately 200 g, were obtained from the Department of Anatomy, Institute of Biosciences/Campus of Botucatu, UNESP—São Paulo State University, Botucatu, Brazil. All animals were individually housed in polypropylene cages (43 × 30 × 15 cm) with autoclaved pine shavings as substrate, and maintained under constant room temperature (23 ± 1 °C) and lighting conditions (12-h light/dark cycle, with the lights switched on at 06:00 h). The animals were provided with a solid diet consisting of Nuvital^®^ (Curitiba, Brazil) rodent chow and filtered water ad libitum.

### 4.2. Experimental Design

Initially, the animals were divided into two groups (*n* = 30): EtOH group, in which the rats had free access to a 10% (*v*/*v*) ethanol solution or water ad libitum, and a control group, composed of rats with no access to ethanol solution. After reaching 65 days of age, the animals had two bottles containing either ethanol solution or water for free choice over a period of 15 days. The animals showing EtOH intake ranging from 2–3 g of ethanol/kg/day were considered to the study [58,59].

When the rats were 80 days old, they were chemically induced with the carcinogen 7,12-dimethylbenz(a)anthracene (DMBA; Sigma Chemical Co., St. Louis, MO, USA), via single injection into the ovarian bursa. During the next 120 days the rats were monitored for OC development by ultrasonography, and ovaries size was used as a representative parameter. After OC development (260-days-old), half of the animals received melatonin (M-5250, Sigma-Aldrich, St. Louis, MO, USA) intraperitoneally at dose of 200 µg/100 g body weight dissolved in small amount of EtOH solution (0.04 mL), and then added to 0.3 mL of 0.9% physiological saline (vehicle) at a final concentration of 0.3 mg/mL. The vehicle was previously tested and had no functional significance at the level of the rat ovary [60]. The daily injections were administered at night (19–19:30 h) over the period of 60 days [61].

Finally, the animals were divided into four groups (*n* = 15): OC group: DMBA-induced animals that did not consume EtOH (vehicle only); OC + Mel group: DMBA-induced animals that received melatonin as therapy; OC + EtOH group: DMBA-induced animals that consumed 10% EtOH solution during OC development and received vehicle; and OC + EtOH + Mel group: DMBA-induced animals that consumed 10% EtOH solution during OC development and received melatonin as therapy. The average EtOH consumed (mL/100g b.w. per day) was 7.1 and 7.6 for OC + EtOH and OC + EtOH + Mel groups, respectively. At the end of the study, the animals were carefully anesthetized and euthanized near the end of the dark period for OC tissue and blood collection. The present experimental protocol was accepted by the Ethical Committee of the Institute of Bioscience/UNESP (CEEA) (# 382, 3 December 2015, Botucatu, Brazil).

### 4.3. Intraovarian Injection of DMBA for Tumor Initiation

After selection for voluntary ethanol consumption, the animals (*n* = 60) were anesthetized with ketamine hydrochloride (60 mg/kg, i.p.) and xylazine (5 mg/kg, i.p.), and a 2-cm incision was made through the skin and muscles for accessing the left ovaries. The left ovarian bursa was identified and the space filled with a single injection of 100 µg DMBA (Sigma Chemical Co.) diluted in 10 µL sesame oil, used as the vehicle [62]. After moving the ovaries back to the cavity, the muscle layer and skin was closed using a 3-0 silk suture. Concomitantly, the right ovary underwent sham-surgery (control ovary) using only the vehicle. All animals received prophylactic treatment with antibiotic (10^5^ units of benzylpenicillin potassium via i.p.). Over the next days, OC development was observed by ultrasonography.

### 4.4. Melatonin Levels

After plasma collection, melatonin was extracted (*n* = 15 samples/group) using HPLC-grade methanol followed by separation on columns (Sep-Pak Vac C-18, reverse phase, 12.5 nm; Water Corporation, Milford, MA, USA). Fifty microliters of samples were used and assayed in a Coat-a-count Melatonin ELISA Kit and read at 405 nm. The samples were run in duplicate and the coefficient of variation was 4%. All reagents and microtiter plates were purchased from IBL (IBL International, Hamburg, Germany), and the concentrations were given in pg/mL.

### 4.5. Histopathology 

Following euthanasia, the ovaries were individually collected and immediately fixed in 10% (*v*/*v*) buffered formalin for 24 h. After fixation, OC tissues were washed and dehydrated in graded ethanol, diaphanized in xylene and embedded in paraplastic (Oxford Labware, St. Louis, MO, USA). 5-μm-thick sections were made using a Leica RM 2165 microtome and every 20th section was stained with hematoylin and eosin (H&E). Histopathological evaluation was carried out by a pathologist with expertise in animal malignancies and only serous papillary carcinoma was considered for further analysis.

### 4.6. Vascular Density

The microvessel density (MVD) was detected by H&E staining. Five random “hot spots” with high concentration of vessels were identified in each slides (*n* = 15 animals/group) and positive areas were counted in a double fashion condition. Total histological area (mm^2^) was considered, and MVD was calculated as previously reported [63].

### 4.7. Immunofluorescence

OC tissues were washed in PBS (phosphate-buffered saline), sodium chloride, potassium chloride, dihydrogen phosphate, and disodium hydrogen phosphate), followed by fixation in 4% paraformaldehyde for 10 min, and permeabilization with PBS. After blocking nonspecific binding sites with 1% bovine serum albumin (BSA), samples were incubated with anti-MT1 primary rabbit polyclonal antibody (dilution 1:100, overnight at 4 °C) followed by secondary polyclonal anti-rabbit IgG conjugated to FITC (1:250, sc-2012, Santa Cruz Biotechnology Inc., Santa Cruz, CA, USA) for 1 h. DAP1 was used for nuclei staining (5 min) at room temperature. For negative immunolabeling, the primary antibody was omitted. Immunopositive reactions were analyzed using a fluorescence microscope (Zeiss Axiophot II, Oberkochen, Germany) at 40× magnification (emission filter at 650 nm for FITC and emission filter at 485 nm for DAPI staining). The fluorescence analysis in merged images was performed using the Image J software (NIH) (Bethesda, MD, USA).

### 4.8. Immunohistochemistry

For immunohistochemistry, sections of OC (*n* = 7/group) were deparaffinized based on the areas previously identified during the histopathology analysis. Antigen retrieval was performed in a microwave for 15 min (3 × 5 min) using 0.01 M sodium citrate buffer, pH 6.0. After endogenous peroxidase was blocked, the tissues were incubated with 3% BSA for 1 h. Then, OC sections were incubated overnight with primary antibodies (Abcam, Cambridge, UK: TGFβ1, VEGF, VEGFR1, VEGFR2, HIF-1α) at dilution 1:100. After immunoreactions, TBS-T buffer was used to wash the sections followed by another incubation with polymer Anti-Mouse IgG or Anti-Rabbit—DAKO^®^ CYT, DAKO Corporation, Carpinteria, CA, USA) for 1 h. The chromogen diaminobenzidine (DAB; Sigma, St. Louis, MO, USA) was added to slides for 5 min, and sections were finally counterstained with hematoxylin. Negative controls were obtained by omitting the primary antibody. IHC analyses were performed under a Zeiss Axiophot II microscope (Carl Zeiss, Oberkochen, Germany) based on the levels of staining intensity (absent 0, weak +, moderate ++ and strong +++).

### 4.9. Western Blot

One hundred milligrams of OC samples (*n* = 7) were rapidly frozen in liquid nitrogen and stored at −80 °C. Only OC tissues containing serous papillary tumors were homogenized with RIPA lysis buffer (Pierce Biotechnology, Rockford, IL, USA) supplemented with protease inhibitors. The homogenates were centrifuged at 21,912× *g* for 20 min at 4 °C to remove cell debris, and total protein was measured through Bradford colorimetric method. After 70 µg protein was added to 1.5× Laemmli buffer, individual samples were loaded per well and resolved into 4–12% acrylamide gradient gels (Amersham Biosciences, Uppsala, Sweden) for 2 h at 60 mA. After electrophoresis, proteins were electrotransferred to nitrocellulose membranes for 1.5 h at 200 mA. Pre-stained standards (Bio-Rad, Hercules, CA, USA) were used as molecular weight markers. Then, the membranes were blocked with TBS-T solution containing 3% BSA for 60 min followed by incubation with the following antibodies: anti-TGFβ1, anti-VEGF, anti-VEGFR1, anti-VEGFR2, anti-HIF-1α (Abcam, Cambridge, UK; 1:1000 dilutions in 1% BSA). The membrane was washed in basal solution (1% TBS-T) and incubated with secondary antibody conjugated with HRP (1:10,000 dilution; Sigma, St. Louis, MO, USA) for 1 h. Thereafter, blot signals were developed using an ECL detection kit (Thermo Fisher Scientific, Rockford, IL, USA). Bands were obtained from individual blots of seven animals/group using image J analysis software (NIH). Β-actin was used as positive control, and data were expressed as the mean ± SD. Immunoblotting were represented as optical densitometry index (% band intensity).

### 4.10. Statistical Analysis

All data were analyzed using one-way ANOVA, and results were expressed as the mean ± SD. Significant differences were calculated with the Tukey test, and statistical level was set at *p* < 0.05. GraphPad Prism 5.0 (La Jolla, CA, USA) software was used.

## 5. Conclusions

In summary, the present study investigated the cross-talk between the VEGF/VEGFR/HIF-1α system and melatonin therapy in an ethanol-preferring rat model of ovarian carcinoma. Melatonin significantly reduced OC mass and microvessel density, thus limiting the potential aggressiveness of serous papillary OC. Furthermore, melatonin therapy exerted anti-angiogenic effects by reducing angiogenic factors (TGFβ1, VEGF, and VEGFR2) related to malignancies in OC, regardless of ethanol consumption. In ethanol-drinking animals, the high levels of HIF-1α were significantly reduced following melatonin therapy. Overall, our findings indicate that melatonin’s regulatory signaling is mediated via its receptor MT1, suggesting melatonin as an adjuvant strategy against angiogenesis in OC.

## Figures and Tables

**Table 1:** Analysis of the immunohistochemical and fluorescence staining.

Target Proteins	Treatments			
	OC	OC + Mel	OC + EtOH	OC + EtOH + Mel
MT1	+	++	+	++
TGFβ1	+++	+	+++	++
VEGF	++	+	+++	+
VEGFR1	+/++	+/++	+	0/+
VEGFR2	+++	+	+++	0/+
HIF-1α	++	+	++/+++	+

Visual OC scoring was evaluated by a pathologist. Representative score was as 0 (no signal), + (weak signal), ++ (moderate signal), or +++ (strong signal). *n* = 7 animals/group. Five OC sections per animal were randomly chosen. MT1: type 1 melatonin receptor; TGFβ1: transforming growth factor-β1; VEGF: vascular endothelial growth factor; VEGFR1: vascular endothelial growth factor receptor 1; VEGFR2: vascular endothelial growth factor receptor 2; HIF-1α: hypoxia-inducible factor 1α.
