# Correction: Zonta et al. Melatonin Reduces Angiogenesis in Serous Papillary Ovarian Carcinoma of Ethanol-Preferring Rats. *Int. J. Mol. Sci.* 2017, *18*, 763

**DOI:** 10.3390/ijms26115150

**Published:** 2025-05-28

**Authors:** Yohan Ricci Zonta, Marcelo Martinez, Isabel Cristina C. Camargo, Raquel F. Domeniconi, Luiz Antonio Lupi Júnior, Patricia Fernanda F. Pinheiro, Russel J. Reiter, Francisco Eduardo Martinez, Luiz Gustavo A. Chuffa

**Affiliations:** 1 Department of Anatomy, Institute of Biosciences, São Paulo State University (UNESP), Botucatu-SP 18618-970, Brazil; yohanzonta@hotmail.com (Y.R.Z.); rdomeniconi@ibb.unesp.br (R.F.D.); lupijr@ibb.unesp.br (L.A.L.J.); pinheiro@ibb.unesp.br (P.F.F.P.); martinez@ibb.unesp.br (F.E.M.); 2 Department of Morphology and Pathology, Universidade Federal de São Carlos (UFSCar), São Carlos-SP 13565-905, Brazil; martinez@ufscar.br; 3 Department of Biotechnology, School of Sciences, Humanities and Languages, São Paulo State University (UNESP), Assis-SP 19806-900, Brazil; camargo@assis.unesp.br; 4 Department of Cellular and Structural Biology, University of Texas Health Science Center at San Antonio (UTHSCSA), San Antonio, TX 78229, USA; reiter@uthscsa.edu

In the original publication [1], there was a mistake in Figures 4 and 5 as published. Unintentionally, the representative images of ovarian cancer in the experimental group treated with ethanol (OC + EtOH) for TGFβ1 and melatonin (OC + Mel) for VEGF and VEGFR1/2 were incorrectly selected in the original manuscript. The authors apologize for any inconvenience caused and state that the scientific conclusions are unaffected. This correction was approved by the academic editor. The original publication has also been updated. The correct Figure 4 and Figure 5 appear below.

## Figures and Tables

**Figure 4:**
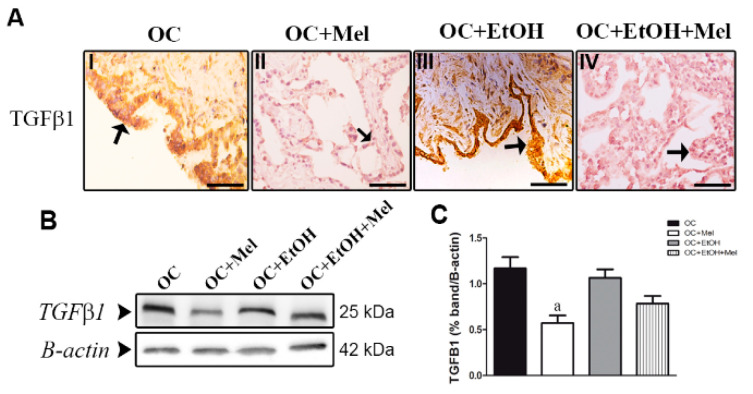
Immunohistochemical localization and western blot analysis of TGFβ1 in serous papillary OC. (**A**) The immunoreaction of TGFβ1 in the epithelial cells of OC was stronger in the OC (**I**) and OC + EtOH (**III**) groups compared to a weak reaction observed in OC + Mel (**II**) and OC + EtOH + Mel (**IV**) animals (black arrows). Bar = 20 µm. Negative controls were used. (**B**) Representative TGFβ1 profile of extracts (70 µg proteins) pooled from seven samples/group (left panel). (**C**) Extracts obtained from individual animals were used for densitometric analysis of the proteins following normalization to house-keeping protein β-actin. All results are expressed as the mean ± SD (*n* = 7). ^a^ *p* < 0.05 vs. OC.

**Figure 5:**
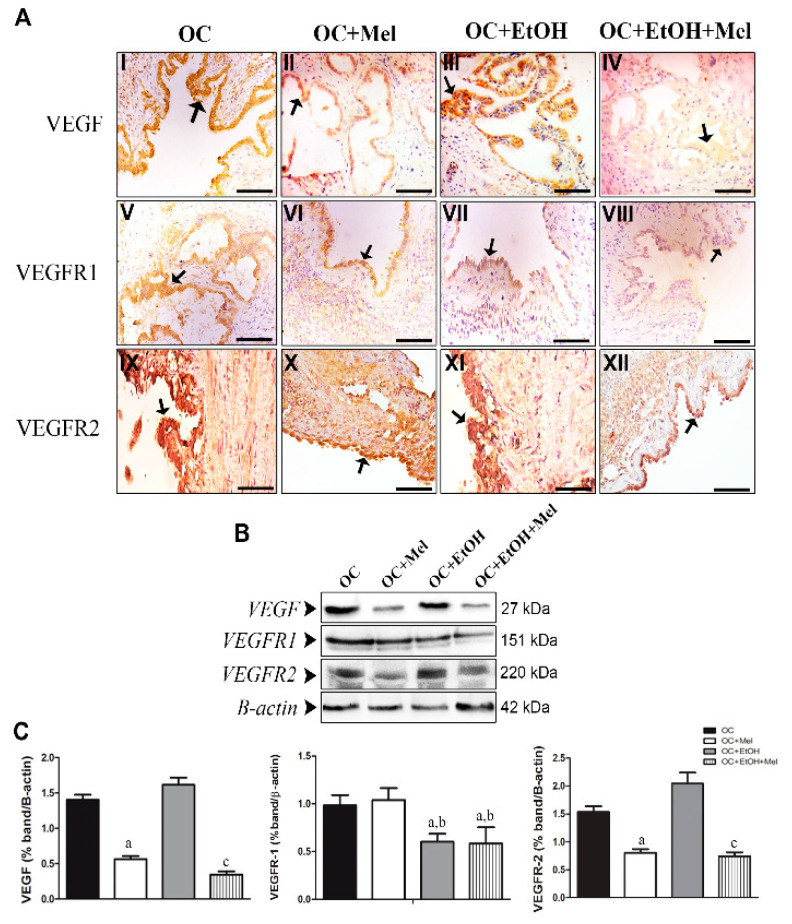
Immunohistochemical localization and western blot analysis of VEGF, VEGFR1, and VEGFR2 in serous papillary OC. (**A**) The immunoreaction of VEGF was moderate to high in the OC (**I**) and OC + EtOH (**III**) groups, while the groups OC + Mel (**II**) and OC + EtOH + Mel (**IV**) showed a weak reaction after melatonin treatment (black arrow). The immunoreactions of VEGFR1 varied from weak to moderate in the surface epithelium of the OC (**V**) and OC + Mel (**VI**) animals, and only a weak reaction was notable in the OC + EtOH (**VII**) and OC + EtOH + Mel (**VIII**) groups (black arrow). A strong reaction to VEGFR2 was present in the papillae epithelium of the OC (IX) and OC + EtOH (**XI**) groups, and melatonin treatment led to weak or even absence of VEGFR2 immunostaining in the OC + Mel (**X**) and OC + EtOH + Mel (**XII**) animals (black arrows). Bar = 20 µm. Negative controls were used. (**B**) Representative profile of the VEGF, VEGFR1, and VEGFR2 levels obtained from protein extracts (70 µg) pooled from seven samples/group (upper panel). (**C**) Extracts obtained from individual animals were used for densitometric analysis of the proteins following normalization to house-keeping protein (β-actin). Data are expressed as the mean ± SD (*n* = 7). ^a^ *p* < 0.05 vs. OC; ^b^ *p* < 0.05 vs. OC + Mel; ^c^ *p* < 0.05 vs. OC + EtOH.
